# Hypothalamic Expression and Moonlight-Independent Changes of *Cry3* and *Per4* Implicate Their Roles in Lunar Clock Oscillators of the Lunar-Responsive Goldlined Spinefoot

**DOI:** 10.1371/journal.pone.0109119

**Published:** 2014-10-01

**Authors:** Riko Toda, Keiko Okano, Yuki Takeuchi, Chihiro Yamauchi, Masato Fukushiro, Akihiro Takemura, Toshiyuki Okano

**Affiliations:** 1 Department of Electrical Engineering and Bioscience, Graduate School of Advanced Science and Engineering, Waseda University, TWIns, Tokyo, Japan; 2 Department of Chemistry, Biology, and Marine Science, Faculty of Science, University of the Ryukyus, Okinawa, Japan; Kent State University, United States of America

## Abstract

Lunar cycle-associated physiology has been found in a wide variety of organisms. Studies suggest the presence of a circalunar clock in some animals, but the location of the lunar clock is unclear. We previously found lunar-associated expression of transcripts for *Cryptochrome3* gene (*SgCry3*) in the brain of a lunar phase-responsive fish, the Goldlined spinefoot (*Siganus guttatus*). Then we proposed a photoperiodic model for the lunar phase response, in which *SgCry3* might function as a phase-specific light response gene and/or an oscillatory factor in unidentified circalunar clock. In this study, we have developed an anti-SgCRY3 antibody to identify SgCRY3-immunoreactive cells in the brain. We found immunoreactions in the subependymal cells located in the mediobasal region of the diencephalon, a crucial site for photoperiodic seasonal responses in birds. For further assessment of the lunar-responding mechanism and the circalunar clock, we investigated mRNA levels of *Cry3* as well as those of the other clock(-related) genes, *Period* (*Per2* and *Per4*), in *S. guttatus* reared under nocturnal moonlight interruption or natural conditions. Not only *SgCry3* but *SgPer4* mRNA levels showed lunar phase-dependent variations in the diencephalon without depending on light condition during the night. These results suggest that the expressions of *SgCry3* and *SgPer4* are not directly regulated by moonlight stimulation but endogenously mediated in the brain, and implicate that circadian clock(-related) genes may be involved in the circalunar clock locating within the mediobasal region of the diencephalon.

## Introduction

Most organisms have endogenous biological clocks to synchronize their physiological functions with environmental cycles. A circadian clock with a period of approximately 24 h is important to anticipate daily changes in the environment. In vertebrates, oscillation of the circadian clock is supported by the transcription-translation feedback loops of core clock components: the positive transcriptional components CLOCK and BMAL, and negative components CRY and PERIOD [1], [2].

Some animals show reproductive responses synchronizing to the lunar-phase with periods of approximately 29.5 days. Lunar-synchronized spawning has been seen in aquatic organisms like coral [3], [4] and spinefoots [5], [6] living in tropical or subtropical zones. While spawning seems to occur according to a species-specific lunar phase, it is still unclear how the timing is determined. There are two possible mechanisms. Spawning may be determined by the integration of lunar-phase information from an endogenous circalunar clock with multiple signals that are linked to environmental changes such as moonlight. Alternatively, spawning could be more instantaneously triggered by the duration and intensity of moonlight depending on the lunar phase. In the latter case, moonlight information conveyed in a specific circadian phase over the course of the night may be recognized by a mechanism similar to that seen in seasonal photoperiodic responses [7].

In coral and spinefoot species, lunar phase, day-length and seawater temperature might be used as cues for spawning behavior. Seawater temperature is suggested to regulate gonad maturation, while lunar cycle determines particular spawning day [8], [9]. Although the molecular mechanism underlying the timing remains elusive, two groups have independently reported that the mRNA levels of circadian clock components change with lunar phase [10], [11]. Light intensity during a full moon might affect the mRNA levels of *Period* (*SgPer2*) in the Goldlined spinefoot (*Siganus guttatus*) [10] and *Cryptochrome* in the coral *Acropora millepora* [11].

In the diencephalon of the Goldlined spinefoot, mRNA levels of *SgCry3* show no daily variation but do peak at the first quarter moon, the phase of spawning in the lunar cycle [7]. Because the diencephalon, especially the hypothalamic region, is the central site for triggering reproductive response in fishes through the secretion of gonadotropin-releasing hormone (GnRH) [12], the oscillation of *SgCry3* might be relevant to the lunar phase recognition mechanism or the regulation of synchronous reproductive behavior.

In this study, we investigated the localization of SgCRY3 protein in the brain to specify its distribution and evaluate its functional significance in the hypothalamic-pituitary-gonadal (HPG) axis, through which the brain controls gonadal maturation in vertebrates. The localization of SgCRY3 in the mediobasal region of the hypothalamus (MBH) led us to further investigate the mRNA expression profiles of circadian clock genes under modified moonlight conditions (constant darkness throughout the night, dark from sunset to midnight, or dark from midnight to sunrise) to assess models corresponding to the two possible lunar-response mechanisms described above: (1) an endogenous circalunar clock that regulates *SgCry3* mRNA expression or (2) moonlight signals that regulate *SgCry3* mRNA expression directly. In the latter case, *SgCry3* mRNA levels would no longer change under the modified moonlight condition(s). As a result, the *SgCry3* mRNA level still changed in all the conditions, and it is suggested that the expression of *SgCry3* is not directly regulated by moonlight stimulation but endogenously mediated.

## Materials and Methods

### Experimental Fish

This study was a collaboration between Waseda University and the University of Ryukyus and was approved by Institutional Animal Care and Use Committees at both participating institutions (see below). Experimental animal care was conducted under permission from the Committee for Animal Experimentation of the School of Science and Engineering at Waseda University (permission # 2012-A085). Animal experiments including sampling in the field were conducted under permission from Sesoko Station Tropical Biosphere Research Center at the University of the Ryukyus (permission # 120519–120604). Juvenile Goldlined spinefoot fish (0.08–0.15 g) were originally collected from a Minato River mangrove swamp in Okinawa, Japan (Latitude 26.6786389, Longitude 127.8883611) using a fish net at low tide around the new moon period. We confirmed that no specific permissions were required for these locations/activities. We confirmed the present field study did not involve endangered or protected species. The fish were reared in outdoor tanks (capacity: 10 metric tons) with aerated running seawater for 1 or 4 years under a natural photoperiod, natural moonlight, and the water temperature conditions at Sesoko Station, Tropical Biosphere Research Center, University of the Ryukyus, Nago, Okinawa, Japan. Frames with roofs over the tanks protected the fish from rain and wind, without restricting sunlight and moonlight. There is no artificial light possibly reaching the fish inside the tanks. Fish were fed commercial pellets (EP1, Marubeni Nisshin, Tokyo, Japan) daily at 10∶00 h.

Approximately 3- or 4-year-old fish with body weights ranging from 288 to 440 g, and approximately 1- or 2-year-old fish with body weights ranging from 18 to 100 g were collected with a fish net May 21, 2012 for use in the present experiments. During the sampling, sunrise and sunset occurred at approximately 5∶30 and 19∶00, respectively. The fish were taken from the tanks at random and anesthetized with iced seawater in a bucket, then brought to the laboratory within a few minutes under natural light conditions. To minimize suffering, the spinal cord was rapidly cut under anesthesia, and the brain (n = 4, diencephalon and optic tectum) were collected from each fish after at 12∶00 under fluorescent light. The brain samples were kept in RNAlater (Ambion) at 4°C overnight and then stored at −80°C until RNA extraction. The brain samples were fixed in Bouin’s fluid at 4°C for 24 h. The fixed tissues were immersed successively in 10, 20 and 30% sucrose in 0.2 M Na-phosphate (pH 7.4) for over 1 day, embedded in 1∶ 2 = Tissue-Tek(R) O. C. T. Compound (SAKURA Finetek Japan) : 30% sucrose in 0.2 M Na-phosphate (pH 7.4), and frozen.

### Antibody

**Figure 1:**
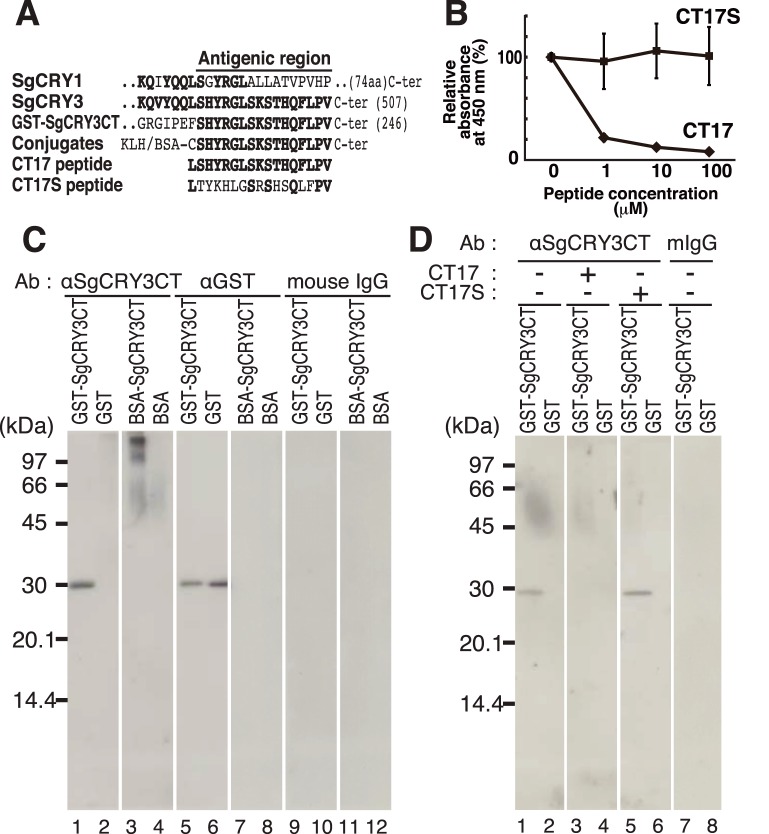
Multiple sequence alignment of the deduced amino acids of *Siganus guttatus* Cryptochrome1 (SgCRY1) and Cryptochrome3 (SgCRY3), competitive ELISA and immunoblot analysis. (A) Multiple sequence alignment of the deduced amino acids of SgCRY1/3. The line above the alignment indicates the antigenic region that was conjugated to KLH or BSA and the conjugate was used as an antigen. CT17 and CT17S are epitope and epitope-shuffled peptides, respectively. (B) Competitive ELISA showing the antigen-specificity. ELISA microplate wells were coated with GST-SgCRY3CT antigen, blocked with 1% skim milk, reacted with αSgCRY3CT that had been mixed with CT17 epitope or CT17S epitope-shuffled peptide at the indicated concentration in advance. Then, the unreacted antibody was washed out and the remaining antibody was detected through use of an HRP-labeled secondary antibody. (C, D) Immunoblot analyses for validating the specificity of αSgCRY3CT to antigenic agent. GST-SgCRY3CT (0.1 µg), GST (0.1 µg), BSA-SgCRY3CT (0.15 µg), and BSA (0.15 µg) proteins were subjected to 10% polyacrylamide SDS-PAGE. In the preabsorption experiment (panel D, lanes 3–6), CT17 or CT17S peptide (100 µM) had been incubated for 1 h at 37°C with αSgCRY3CT before the primary antibody reaction.

A synthetic peptide for carboxyl-terminal 16-amino-acids of *Siganus guttatus* CRY3 (Ser^492^-Val^507^; NH_2_- SHYRGLSKSTHQFLPV-CO_2_H termed SgCRY3CT) with a cysteine residue added at its amino terminal was conjugated to keyhole limpet hemocyanin (KLH), and the conjugate was used as an antigen (Figure 1A). The antigen was injected into mice 5 times and the serum was collected. The serum was diluted with PBS (10 mM sodium phosphate (pH 7.4), 140 mM NaCl, 1 mM MgCl_2_) and purified using the SgCRY3CT-conjugated HiTrap NHS-activated HP column (GE Healthcare). Proteins with specific binding were eluted by elution buffer (0.1 M glycine (pH 2.7)). The eluates were neutralized, dialyzed against PBS, and supplemented with glycerol to 50%. This purified antibody (termed αSgCRY3CT) was stored at −20°C until use [13].

### Immunoblot Analysis

Immunoblot analysis was performed as described previously [14]. The primary antibodies (αSgCRY3CT or anti-GST Antibody (B-14) (SC-138, Santa Cruz Biotechnology)) or control mouse IgG (7056S, Cell Signaling) were used at 1.0 µg/ml.

### Immunohistochemistry

The frozen brain samples were sectioned transversely at 10 µm. The sections were treated with blocking solution for 1 h at room temperature and then incubated 16 h at 4°C with the primary antibody (0.7 µg/ml, αSgCRY3CT or mouse IgG). After washing sections at room temperature, the sections were successively incubated with a biotinylated anti-mouse IgG and avidin-biotin complex solution using the Vectastain Elite ABC kit (Vector Laboratories). Positive signals were visualized by incubating the slides for 15 min in TBS (50 mM TrisHCl (pH 7.4), 200 mM NaCl, 1 mM MgCl_2_) containing 0.1% diaminobenzidine and 0.02% H_2_O_2_.

### Moonlight Conditions

One- or two-year-old fish (∼170) were divided into 4 groups, each of which was kept in a 200 L tank. By covering each tank with a blackout sheet and removing it daily, the lighting of the three experimental tanks could be controlled for constant darkness throughout the night (DD, dark and dark conditions), dark from 30 minutes after sunset to midnight (DM, dark and natural conditions), or dark from midnight to 30 minutes before sunrise (MD, natural and dark conditions). The control tank was kept under natural moonlight throughout the experiments (MM, natural and natural conditions).

### Quantitative RT-PCR Analysis

Total RNA was extracted from the tissues using TRIzol reagent (Life Technologies). Residual genomic DNA in the total RNA sample was eliminated by DNase I treatment (RNase-free recombinant DNase I, TaKaRa BIO). Quantitative RT-PCR analyses were performed using StepOnePlus (Applied Biosystems) along with a high capacity cDNA reverse transcription kit (Applied Biosystems) and Fast SYBR Green Master Mix (Applied Biosystems). Each reaction included 1 µg of total RNA as a template. The primers for quantitative RT-PCR are shown in Table 1 [7], [10], [15]. The reference control gene was virtually defined as the average of the threshold cycles (Ct) for *SgPGK, SgEF1α* and *Sgβ-actin* as reported in Vandesompele *et al.* (2002) [16].

**Table 1:** Primers for quantitative RT-PCR.

Gene name	Primer name	Sequence
*SgCry3*	SgCry3 RT_PCR_F	GGTGTGGAGACTATTGTCAGAAACTCA
	SgCry3 RT_PCR_R	CTTCCAGCGATGGGATACTGTATAAC
*SgPer2*	SgPer2-RTPCR-F	CTGTTGGGTTACCTCCCTCA
	SgPer2-RTPCR-R	AAGCGGATCGAGGAGTGATCA
*SgPer4*	SgPer4-RTPCR-F	CCCTCCAGACAAGAGGATCTTC
	SgPer4-RTPCR-R	CCCCGCAAACTGAAAGATCT
*SgEF1α*	Sg_EF1a_qRT-PCR_F	CACAGGGACTTCATCAAGAACATGATC
	Sg_EF1a_qRT-PCR_R	CGTTCTTGGAGATACCAGCCTC
*SgPGK*	PGK_qRT-PCR_F2	CCTCAAAGTGCTCAACAACATGGAG
	PGK_qRT-PCR_R2	CTCATCGAACTTGTCAGCGGTG
*Sgβ*–*actin*	actin_qRT-PCR_F	CATCGCTGACAGGATGCAGAAG
	actin_qRT-PCR_R	CTCCGATCCAGACAGAGTATTTACG

### Statistical Analysis

Data were analyzed using two-way ANOVA with Tukey-Kramer multiple comparisons on Statcel2 (the add-in forms on Excel (Microsoft)) software.

## Results

### Antibody to SgCRY3

To investigate the cellular localization of SgCRY3 protein in the Goldlined spinefoot, we prepared a polyclonal antibody to the carboxyl terminal hexadecapeptide of SgCRY3, which has little or no sequence homology to SgCRY1, another CRY we had previously identified in this fish [7] (Figure 1A, Antigenic region). After immunoaffinity purification of the antibody from antiserum using an SgCRY3CT-conjugated column, immunoreactivity and specificity of the purified antibody (termed αSgCRY3CT) to the antigenic peptide was verified by competitive ELISA (Figure 1B), immunoblot analysis (Figure 1C, 1D) using the fusion protein GST-SgCRY3CT or BSA conjugated with synthetic SgCRY3CT peptides (Figure 1A). Preincubation of the αSgCRY3CT antibody with the epitope peptide CT17 suppressed the immunoreacition to GST-SgCRY3CT antigen in ELISA in a dose dependent manner (Figure 1B), and this suppression was not observed when CT17S control peptide was used instead of CT17. In the immunoblot analysis, αSgCRY3CT showed specific immunoreactive bands for GST-SgCRY3CT (Figure 1C, lane 1), with mobility of ∼30 kDa, and for BSA-SgCRY3CT, with a higher molecular weight (Figure 1C, lane 3). Being consistent with the result in ELISA, immunoreaction to GST-SgCRY3CT in immunoblot analysis was completely suppressed only in the presence of 100 µM CT17 peptide (Figure 1D, lane 3). There was no immunoreactivity to native proteins in the Goldlined spinefoot tissues (diencephalon, optic tectum, and ovary) most likely due to the low expression level.

### Immunohistochemical Localization of SgCRY3 in the Brain

**Figure 2:**
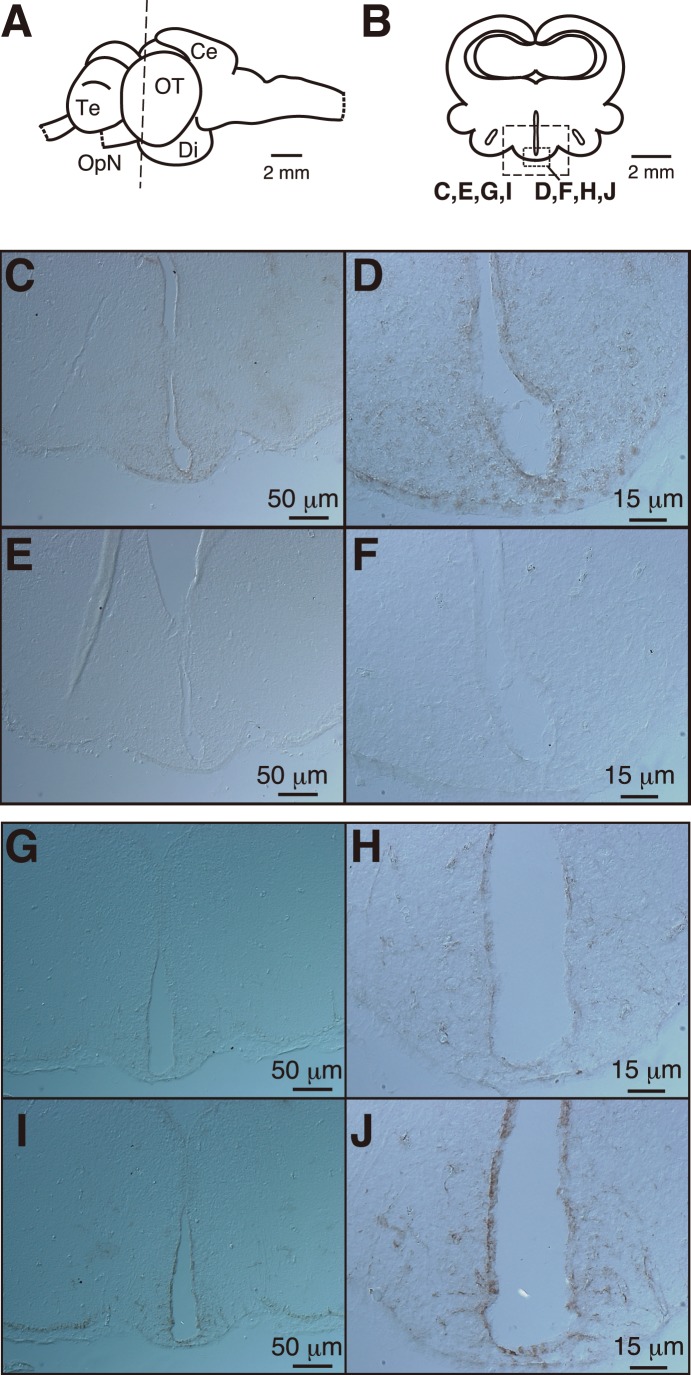
Immunohistochemical localization of *Siganus guttatus* Cryptochrome3 (SgCRY3) in the diencephalon. (A) Drawing of the lateral view of the brain of *Siganus guttatus*. Lettered dotted lines indicate the levels of the transverse sections shown in panels C–J. Ce, cerebellum; Di, diencephalon; OpN, optic nerve; OT, optic tectum; Te, telencephalon. (B) Drawing of the transverse sections at the level of panel A. (C, D) αSgCRY3CT staining without a competitive peptide. (E, F) control sections (mouse IgG was used instead of αSgCRY3CT). (G, H) αSgCRY3CT staining in the presence of 100 µM CT17 epitope peptide. (I, J) αSgCRY3CT staining in the presence of 100 µM CT17S epitope-shuffled peptide. In the preabsorption experiment (panels G–J), CT17 or CT17S peptide (100 µM) had been incubated for 16 h at 4°C with αSgCRY3CT before the primary antibody reaction. Panels D, F, H, and J are magnified view of panels C, E, G, and I, respectively. Wash solution; PBS containing 0.25% (panels C–F) or 0.05% (panels G–J) of Triton X-100. Blocking solution; Wash solution containing 1.5% horse normal serum. Each tissue was sampled either March 23, 2012 (new moon) or June 27, 2014 (new moon).

Immunohistochemical investigation of sections through the brain from the telencephalon to the cerebellum (Figure 2A) revealed that SgCRY3-like immunoreactivities were expressed in the MBH of the diencephalon (Figure 2B, 2C, 2D). These immunoreactions were seen in cell bodies of ependymal cells. The immunopositive cells were found to span a width of approximately 1.5 mm along the anteroposterior axis. Antigen specificity in the immunoreaction was confirmed by the preabsorption experiment (Figure 2G–2J), in which the immunoreaction was significantly weakened by preincubation of the αSgCRY3CT antibody with CT17 peptide (Figure 2G, 2H), while CT17S control peptide had minimal effects (Figure 2I, 2J).

### Lunar Variation of SgCry3 mRNA Expression in the Diencephalon and Optic Tectum

**Figure 3:**
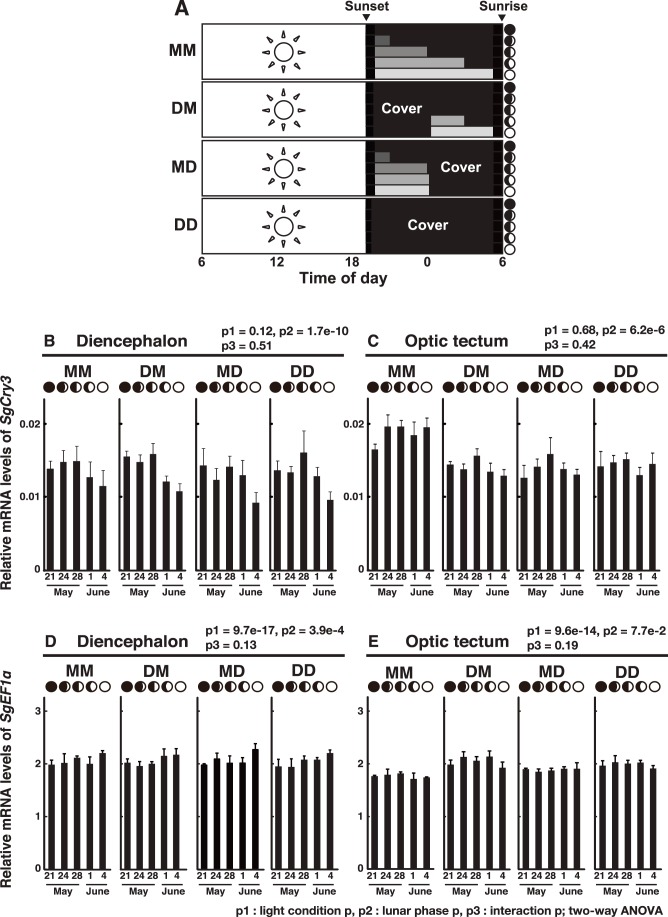
Lunar phase-dependency of *Siganus guttatus Cryptochrome3* (*SgCry3*) mRNA expression in the brain. (A) Experimental design using a tank cover for nocturnal moonlight interruption. Four groups of fish were contained in tanks maintained under natural (MM, natural and natural condition) conditions or constant darkness from 30 minutes after sunset to midnight (DM, dark and natural condition) or constant darkness from midnight to 30 minutes before sunrise (MD, natural and dark condition) or constant darkness from 30 minutes after sunset to 30 minutes before sunrise (DD, dark and dark condition) from May 21 (new moon) to June 4 (full moon). Illustration shows nocturnal light conditions in tanks and the time of moonlight irradiation from May 21 to June 4, 2012. Lunar phases are indicated by schematic moon images. (B–E) Lunar changes in *SgCry3* mRNA levels in the brain. The diencephalon and optic tectum (n = 4) were collected at noon from the new moon to full moon phase. *SgCry3* mRNA levels were calculated as values relative to those of the virtual reference control gene and were defined as the average of the threshold cycles (Ct) for *SgEF1α*, *SgPGK* and *Sgβ-actin.* Error bars represent ± SD. The p values are indicated on each graph, two-way ANOVA.

Because moonlight (∼0.7 lx) decreases plasma melatonin concentration in the Goldlined spinefoot [17], this fish may utilize a phase-dependent moonlight stimulus to detect the moon phase. If the fish use moonlight duration or intensity (Figure 3A) to detect the moon phase by a photoperiodic mechanism, *SgCry3* mRNA levels would no longer change when the moonlight signals were eliminated. On the other hand, if expression is regulated by an internal oscillator, the *SgCry3* mRNA level would still change in the absence of moonlight.

On the basis of these considerations, four groups of fish were repeatedly exposed to different nocturnal moonlight regimes through the use of blackout sheets to cover the tanks (Figure 3A, MM, DM, MD, and DD) to investigate the effects on lunar phase-dependent variation in *SgCry3* mRNA levels. Tissue samples of the diencephalon and optic tectum were taken from each fish at noon to estimate the mRNA levels of *SgCry3*, *SgPer*s (see below), and control genes. The optic tectum was selected as a control tissue out of the diencephalon to decipher whether the *SgCry3* mRNA variation would be isolated to the diencephalon. In all groups except for group MM, *SgCry3* mRNA levels in the diencephalon significantly decreased from the first quarter moon (May 28) to the full moon (June 4) (p<0.01 in DM, p<0.05 in MD and DD; Tukey-Kramer post hoc test) (Figure 3B). A similar tendency was also observed in the MM group, and there is no significant difference among the four groups (p = 0.12; two-way ANOVA), implicating that the light treatments have little or no effect on the *SgCry3* mRNA levels in the diencephalon. The variation profile in *SgCry3* mRNA levels correlated well with our previous observations under natural conditions [7].

In the optic tectum, averaged levels of *SgCry3* mRNA expression were comparable to those in the diencephalon, but a lunar phase-dependent variation in *SgCry3* mRNA levels was not detected (Figure 3C), although the mRNA levels in group MM were slightly higher than the other groups (p<0.01, MM vs DM, MD, DD; Tukey-Kramer post hoc test). In one of the control genes, *SgEF1α* (Figure 3D, 3E), mRNA levels showed little or no lunar phase-dependent variation in the diencephalon or optic tectum.

### Lunar Variations of SgPer2 and SgPer4 mRNA Expression in the Diencephalon

**Figure 4:**
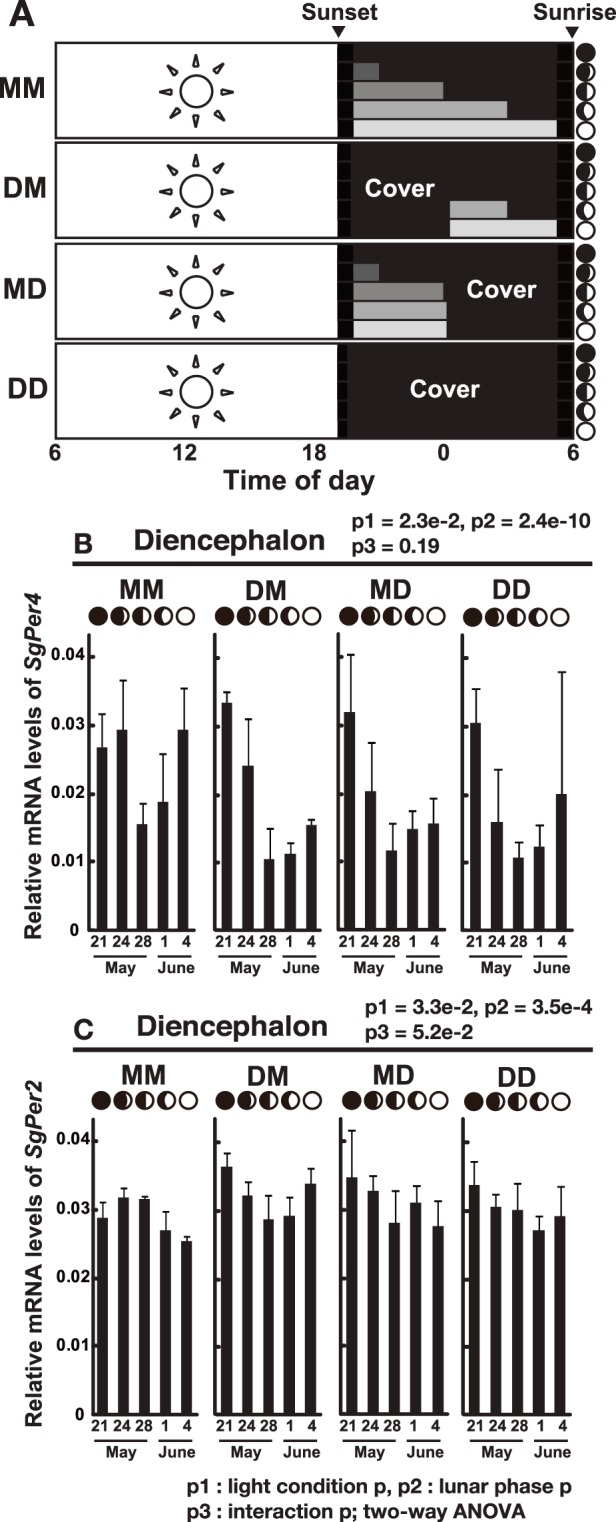
Lunar phase-dependency of *Siganus guttatus Period4* (*SgPer4*) and *Period2* (*SgPer2*) mRNA expression in the brain. (A) Experimental design using a tank cover for nocturnal moonlight interruption. See legend to Figure 3A. (B, C) The diencephalon (n = 4) was collected at noon from the new moon to full moon phase. *SgPer4* (panel B) and *SgPer2* (panel C) mRNA levels were calculated as values relative to those of the virtual reference control gene and were defined as the average of the threshold cycles (Ct) for *SgEF1α*, *SgPGK and Sgβ-actin.* Error bars represent ± SD. Lunar phases are indicated by schematic moon images. The p values are indicated on each graph, two-way ANOVA.

*Period* genes (*Per2* and *Per4*) have also been identified as clock or clock-related genes in the Goldlined spinefoot [10], [15]. *Per* mRNA levels show circadian rhythms with high amplitude in most clock cells, and PER proteins play an important role in the circadian negative feedback loop that is achieved by PER:CRY heterodimers [1], [2]. We hypothesized that CRYs work in cooperation with PER in the hypothalamus, and hence investigated lunar phase-dependent variation in mRNA levels of *SgPer4* and *SgPer2* (Figure 4). Interestingly, *SgPer4* mRNA levels showed lunar-dependent changes in all the experimental groups (Figure 4B). They were lowest on May 28 (first quarter moon), and ratios for the maximum to the lowest levels were 1.9 times (MM) ∼ 3.2 times (MD) (p<0.01 in DM and MD, p<0.05 in MM; Tukey-Kramer post hoc test). On the other hand, *SgPer2* mRNA levels showed no or only minimal changes over the experimental period in all the groups (Figure 4C).

## Discussion

Our previous investigation of the lunar phase-dependent expression of *SgCry3* mRNA in the diencephalon [7] prompted us to obtain more precise information for localization of SgCRY3 protein in the brain in order to clarify the biological relevance of SgCRY3. Interestingly, cells with SgCRY3-like immunoreactivities were detected in the MBH (Figure 2C, 2D), the crucial site for regulation of the photoperiodic gonadal maturation in quails [18]–[21]. In the sapphire devil (*Chrysiptera cyanea*), a tropical fish, photoreceptors in the MBH that regulate the day length-dependent ovarian maturation have been implied [22]. In the present study, we believe the ependymal cells in the MBH are SgCRY3-like immunoreactive (Figure 2C, 2D). These cells are reminiscent of cerebrospinal fluid (CSF)-contacting neurons in deep brain regions of the pigeon and toad, which are implied to integrate photic and circadian signals [23], [24]. In theory, the SgCRY3-immunopositive cells could be receptive to lunar phase and control the HPG axis through the integration of environmental moonlight and circalunar and/or circadian clock information. Due to the limited number of available fish in the present study, we did not analyze lunar phase-dependent variation in SgCRY3 protein levels and localization. Further study on the spatiotemporal changes of SgCRY3 and *in vitro* molecular analyses may be needed to determine the physiological role(s) of SgCRY3.

When four groups of *S. guttatus* were exposed to different nocturnal lighting conditions (Figure 4A), *SgPer4* mRNA expression decreased during the first quarter moon regardless of the light conditions (Figure 4B) and most likely depended on *SgPer4-*specific transcriptional control. Based on the present results together with the previous observation that mRNA levels of *SgPer4* show daily change in the brain and pineal [15], *SgPer4* expression may be under a combined regulation from both daylight and moonlight variations much like *SgCry1*, the mRNA level of which changes in both a daytime- and lunar phase-dependent manner [7].

In our previous study [7], we speculated that the lunar phase-dependent variation in *SgCry3* mRNA expression might be governed by a mechanism similar to a photoperiodic response; the moonlight stimulus during late night from the first quarter moon to full moon may repress the expression of *SgCry3*. Alternatively, the lunar response may be directly regulated by an endogenous lunar clock, which is entrained via a phase resetting pathway by an external signal(s) such as cyclic moonlight stimuli. The present study was undertaken to investigate which mechanism might occur in *S. guttatus*. Considering the results from *SgCry3* (Figure 3B) and *SgPer4* (Figure 4B), the latter case seems to be more plausible. That is, mRNA levels of these two genes are likely regulated not by a photoperiodism-like mechanism directly but rather an endogenous lunar-associated signal, although we cannot fully deny the possibility that they are regulated directly by a non-photic signal with lunar phase-dependent variation such as geomagnetic activity [25] or coral-originated estradiol in sea water [26]. The presence of a lunar clock has been confirmed in an invertebrate species [27], but has yet to be elucidated in vertebrate species. The present data strengthen the presence of an endogenous lunar clock in *S. guttatus*, an idea that is highly consistent with previous observation of the synchronous spawning that occurs in *S. guttatus* even when reared under constant nocturnal darkness from 2 weeks before the expected spawning date [17].

It should also be noted that the expression profiles for *SgCry3* and *SgPer4* are quite different. This indicates that *SgCry3* and *SgPer4* fulfill criteria for state variables in that they define the lunar phase by a combination of their activity levels. Together with our own recent findings and those of other investigators [7], [10], [11], [27], the present data underpin intimate molecular links that overlap between circadian and lunar clock systems. Further investigation of circadian clock components in *S. guttatus,* with special attention paid to the deep brain regions, would be important to reveal the molecular mechanism underlying the lunar-response systems.
